# The Use of Social Media to Recruit Participants With Rare Conditions: Lynch Syndrome as an Example

**DOI:** 10.2196/resprot.6066

**Published:** 2017-01-23

**Authors:** Allison M Burton-Chase, Wendy M Parker, Kelsey Hennig, Faith Sisson, Linda L Bruzzone

**Affiliations:** ^1^ Albany College of Pharmacy and Health Sciences Albany, NY United States; ^2^ Advocacy Consultant Vacaville, NY United States

**Keywords:** social media recruitment, Lynch syndrome, participation rates, response rates, data collection

## Abstract

**Background:**

Social media is increasingly being used as a means of recruiting participants, particularly for investigators whose areas of interest involve rare conditions or hard-to-reach populations. However, much of the literature to date has focused on paid advertisement recruitment.

**Objective:**

We used Lynch syndrome (LS), a rare hereditary cancer syndrome, as a model to demonstrate the successful partnership between researchers and a Web-based patient education and advocacy organization to facilitate participant recruitment.

**Methods:**

Recruitment was undertaken in partnership with Lynch Syndrome International (LSI), an advocacy organization with a strong social media presence. After LSI published our study information, participants followed up via email or phone call. Following prescreening and consent, interested and eligible participants were then sent a secure survey link.

**Results:**

Within 36 hours of a single Facebook post by the site administrators for LSI, over 150 individuals responded via phone or email. Sixty-five individuals were sent the survey link and 57 individuals completed the survey (88% response rate). Of note, these 57 individuals were geographically diverse within the Unites States, representing LS patients from 26 different states.

**Conclusions:**

This approach has several advantages, including recruitment through a trusted source outside of a clinical setting, higher response rates, and cost-effectiveness with a small research team in a relatively short amount of time. Overall, social media recruitment with a trusted online partner can be highly effective in hard-to-reach clinical populations, such as patients with LS. However, this approach requires additional effort for eligibility screening.

## Introduction

Historically, investigators with an interest in rare illnesses or hard-to-reach populations have relied on clinics or hospitals for access to potential research participants. However, individuals have become increasingly interested in using the Internet to seek health information, making social media a highly valuable recruitment tool. Patients, especially those with rare conditions, often use the Internet to build communities, find information, and establish a support network [1,2]. Facebook alone has an average of 1.09 billion daily users, and many patient education and advocacy organizations maintain an active social media presence [3,4]. Social media recruitment has been shown to increase accessibility of target populations along with willingness to participate in research [5]. Social media also has the potential to reach understudied and demographically-diverse populations at a higher yield compared to traditional recruitment methods [6].

Current literature indicates that social media and electronic medical records continue to expand the ways in which patients and providers connect with each other and/or communicate regarding health care decisions [7]. However, social media recruitment, as currently documented in the literature, largely focuses on paid advertisements that attempt to recruit respondents directly to an online survey [8,9]. Additionally, there are some established concerns regarding social media recruitment (specifically privacy, access, security, cost, and the gathering of patient-reported outcomes) that are less of a concern in our study [10]. We recruited people who are actively engaged in social media that focuses on Lynch syndrome (LS), making both privacy and access to online communities less challenging. Security concerns that are documented in the existing literature are less relevant for this project, as the post on the Facebook page of Lynch Syndrome International (LSI; our community partner) provided us only with names and contact information [11]. Individuals responded to the Facebook post, and research staff were then responsible for screening participants for eligibility, obtaining consent, and sending a secure link to an anonymous survey. There were no costs associated with our recruitment methodology, as our Facebook post was free and the survey was security-enabled via Research Electronic Data Capture (REDCap [12]; explained in more detail below). Notably, our procedural differences in social media recruitment may result in a study population that is similar to existing study samples in hereditary cancer research (eg, more affluent, higher educated, and white). The primary difference between participants recruited through clinic-based methods and those recruited through social media was the inclusion of patients who were receiving care outside of major cancer centers when recruitment was completed through social media.

In this paper, we use LS, a rare hereditary cancer syndrome that predisposes an individual to a variety of cancers (including, but not limited to, colorectal, endometrial, and ovarian cancer [13,14]), as a model for successfully partnering with a Web-based patient education and advocacy organization for participant recruitment. We outline study details that can be replicated in other studies.

## Methods

**Figure 1:**
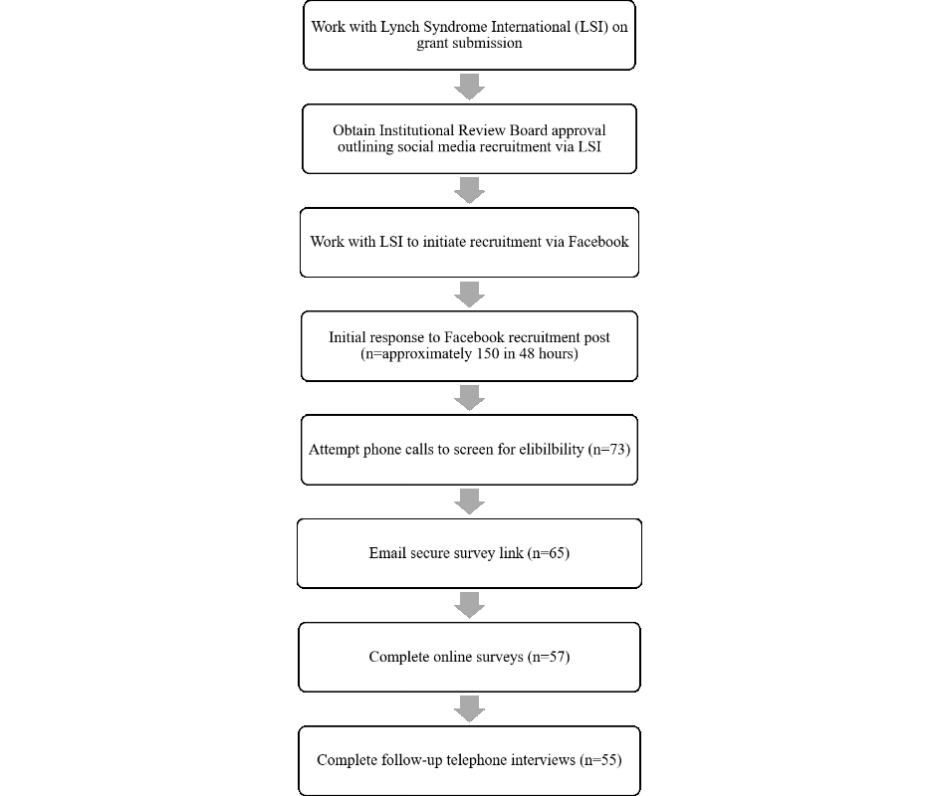
Recruitment process and outcomes.

This study was approved by the Institutional Review Board of the Albany College of Pharmacy and Health Sciences. The entire recruitment process and outcomes are outlined in Figure 1. To be eligible for the study, individuals had to meet the following criteria: (1) at least 18 years of age, (2) able to read and speak English, (3) completion of genetic testing for an LS mutation; and (4) diagnosis of an LS mismatch repair mutation. Participants were recruited through LSI, a patient education and advocacy organization whose mission is to serve the LS community by providing support for families affected by this diagnosis, creating public awareness of the syndrome, educating the public and health care professionals about LS, and providing support for research [15]. The organization is founded and governed by LS survivors, their family members, and health care professionals who specialize in LS, making it a trusted source of information and interaction for LS patients and their families. The LSI website includes accurate and up-to-date diagnosis, treatment, and management information, as well as message boards [15]. LSI is active on social media, including Facebook and Twitter, and members of their community frequently post and respond to questions by other members, and discuss new LS-related research [4]. LSI provided a letter of support for the initial grant application and, prior to participant recruitment, the executive board of LSI reviewed the approved protocol and held a conference call with the Principal Investigator to discuss study aims and the recruitment plan. When all issues were resolved and the study was open for recruitment, LSI posted the announcement on their Facebook page, which established our credibility with the potential study population.

**Figure 2:**
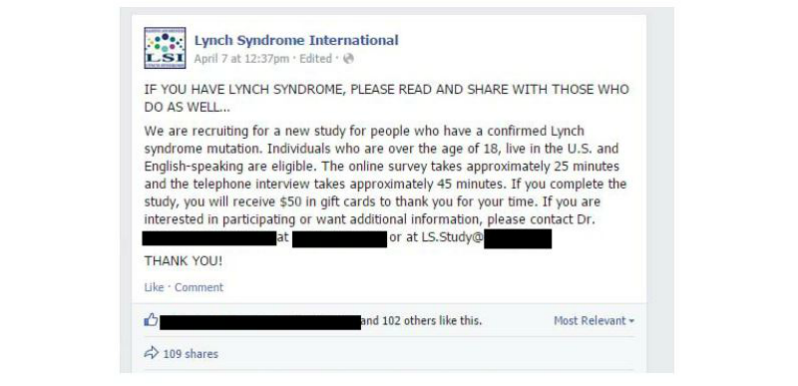
Facebook recruitment post.

**Figure 3:**
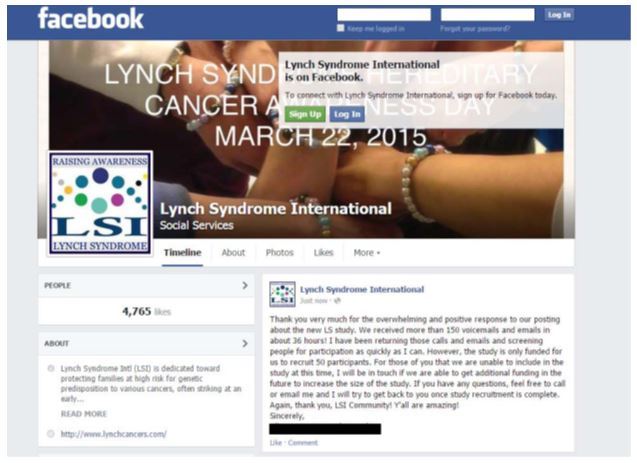
Thank you Facebook post.

A Facebook post (Figure 2) directly from LSI was used for recruitment, and contained eligibility criteria and study details, such as study procedures, time commitment, and incentives. Interested individuals were given instructions to respond either via email or phone. To confirm eligibility, one member of the study team called potential participants to screen for study eligibility in the order that they responded; these calls lasted approximately 5-10 minutes. If the person did not answer, a voicemail was left and the next person on the list was called. After the participant was screened for eligibility, a secure survey link with instructions for completion was sent via REDCap, a browser-based electronic data capture software package, that allowed us to create and manage the online surveys and provided a mechanism for data capture in a quick, secure, Health Insurance Portability and Accountability Act-compliant manner. Our institution has a site license for REDCap and only personnel with approved access can access the survey data, which is held on a secure server that can only be accessed on campus by approved study personnel. Once a survey is initiated or completed in REDCap, the data is stored without being linked to the email address of the individual participant, leaving no identifiable information in the surveys. A separate secure system exists within REDCap that tracks when the survey links are opened and when surveys are completed, but the participant identification number is not linked to that system. All participants who completed the online questionnaire were mailed a US $25 gift card as compensation, and were called to schedule the in-depth, semistructured telephone interview. Participants who completed the qualitative interview were mailed a second US $25 gift card as compensation. Recruitment was discontinued when the survey link was sent to 65 potential participants, as a completion rate of approximately 75% was anticipated for both the survey and interview. This estimated completion rate was based on prior studies by our research team piloting this recruitment method; the estimated completion rate would result in a final sample size of 50. Research staff also maintained a relationship with both LSI and potential respondents by following-up two days after the initial call for participants with a *Thank You* post on Facebook (Figure 3). This post served three purposes. First, it communicated to individuals who had responded that we were systematically working through the responses and contacting potential participants. Second, it let new people who were just seeing the study information for the first time know that we had already surpassed the number of responses for the study sample size, thus decreasing the number of continued responses. Finally, it allowed anyone who was looking at the Facebook page to get a sense of the community response. These small gestures from research staff continue to build relationships, and ensure the possibility of future research, within this community.

## Results

Within 36 hours of a single Facebook post by the site administrators of LSI, over 150 individuals responded via phone or email (Figure 1). One member of the research team (ABC) contacted 73 respondents to reach an anticipated final sample of 55 participants (75% overall response rate). Sixty-five of those individuals were sent the secure link; 57 participants completed the online survey, while 8 individuals did not (88% response rate). Of those 8 individuals, 6 did not finish, 1 did not consent, and 1 never opened the survey link. Follow-up interviews were conducted with 55 of the 57 participants who completed the survey (96% response rate); 2 individuals were not able to be contacted during the study period. As expected, the final sample of 55 participants was primarily female (n=41; 75%) and white (n=51; 93%). However, there was significant geographical diversity within the United States among participants, with 26 states represented in the final sample. There was also heterogeneity in terms of rural and urban communities and regions of the country. Additionally, approximately half of our sample were previvors (n=27; 49%), meaning that they have tested positive for an LS mutation but have not yet had a cancer diagnosis. This split between cancer survivors and previvors will allow us to study group differences in care transitions and provider satisfaction; this comparison is one we have not been able to do in prior studies due the difficulty of recruiting previvors when using traditional clinic-based methods.

The survey link was sent in three batches. The first batch was sent the day after the announcement was posted, the second was sent the following day, and the third was sent 5 days after the Facebook post. The average time for survey completion was 3 days (median=2; range=1-16) after receiving the survey link (responding on the same day was classified as 1 day to complete). The approved protocol stated that the research team would wait 2 weeks after survey completion to contact participants to set up the in-depth telephone interviews. The average number of days between survey completion and participation in the phone interview was 33 (range=21-42). Three members of the research team conducted the telephone interviews. The online survey took approximately 45 minutes to complete, however there was significant variability due to skip patterns and complexity of personal and family health history; the in-depth telephone interviews ranged in length from 25 minutes to 2 hours. Overall, the time from the initial call for participants to the completion of data collection (online survey and phone interview) was 45 days, which was significantly shorter than in prior studies by our research team in the same population.

## Discussion

This study demonstrates the effectiveness of partnering with a patient education and advocacy organization to recruit participants with rare conditions or hard-to-reach populations. This method can be utilized outside of a traditional clinical setting and affords investigators who are not affiliated with major medical centers (and thus, without direct access to clinical populations) the opportunity to conduct valuable research in their target populations. However, the reputation of the community partner is a critical component. While this study did not directly obtain data on reasons for participation, participants did take the opportunity both during the eligibility screening call and telephone interview to informally discuss this topic. During those conversations, many participants cited their confidence in the merits of this study due to its endorsement and publicization by LSI. Respondents trusted that LSI had vetted the study appropriately prior to posting the call for participants. Stated simply, it appears that our recruitment may have been particularly successful because LSI is a respected and trusted organization within the LS population. However, given that these data were not systematically collected, further research is needed to determine how large of a role this factor played in our successful recruitment efforts.

Other advantages of this methodology are that it can be carried out with a small research team in a short amount of time. These factors are highly beneficial for pilot studies with small budgets or tight timelines. Notably, the response rate using social media recruitment was significantly higher than prior studies in the same population [16-18]. This result could be due to the two-step recruitment process, allowing researchers to screen for participants who were eligible while also establishing a personal connection. Posting an open recruitment call allowed individuals to opt-in to the study, and the subsequent telephone call to screen for eligibility provided potential participants with the opportunity to ask questions and gain a greater understanding of the study goals. This level of interaction, along with the validation of the community partner, appears to have increased participant commitment, as evidenced by our high response rate. In addition, in a climate of limited funding for research and the need to document the ability to connect to communities prior to applying for federal grant funding, this technique could be invaluable. Costs were limited to researcher time, incentives, postage, and transcription of the qualitative interviews. The institutional access to a secure online data collection tool was essential, as well as the partnership with LSI.

Researchers who utilize this methodology must be aware of the potential for participant bias. One potential concern is that respondents recruited through social media may not be representative of the entire population, which should be acknowledged when data are published; however, prior research has shown that participants recruited through social media tend to be more demographically diverse than traditional clinical populations [6]. In our experience, the participant demographics of individuals recruited through social media are similar to those recruited through traditional sources for hereditary cancer studies. However, both have been noted to over-represent individuals who are more highly educated, female, and white. In terms of increasing the heterogeneity of the study sample, the use of social media as a recruitment method allows researchers to reach individuals, such as previvors, who are receiving care in the community (as opposed to a single institution or comprehensive cancer center) and in geographically-diverse areas. Although this methodology does not fully address the broader bias in research participation for hereditary cancer populations, which merits continued discussion by researchers and clinicians, it is a step forward in terms of including individuals who would previously have been left out of these types of studies. Finally, concerns regarding privacy and security were procedurally minimized in this study. Patients were open about their health circumstances as part of the LSI online community, surveys were anonymized, and data were securely collected via REDCap. Future research could easily connect to clinical data via electronic health records in this same structure to combine patient-reported outcomes with the depth of clinical data.

### Conclusions

Creating partnerships with Web-based patient education and advocacy organizations and social media recruitment are powerful tools for health researchers, especially those who study hard-to-reach populations. If utilized effectively, this recruitment method can open up research opportunities for investigators, particularly those who do not have direct access to clinical populations or are working with limited staff and budgets. It is also clear that the choice of community partner must resonate and have credibility with the population of interest. Organizations must have both an active social media presence, and be seen as valuable and trusted community resources, by potential participants. LSI provides connections, shares up-to-date health information, and provides education to LS patients; therefore, when a research opportunity was posed to potential participants via the LSI website, respondents trusted that the study has been properly vetted and were willing to share their opinions and experiences.

## Abbreviations

LS: Lynch syndrome
LSI: Lynch Syndrome International
REDCap: Research Electronic Data Capture
